# CPredictor3.0: detecting protein complexes from PPI networks with expression data and functional annotations

**DOI:** 10.1186/s12918-017-0504-3

**Published:** 2017-12-21

**Authors:** Ying Xu, Jiaogen Zhou, Shuigeng Zhou, Jihong Guan

**Affiliations:** 1 Department of Computer Science and Technology, Tongji University, Shanghai, 201804 China; 2 Shanghai Key Lab of Intelligent Information Processing, and School of Computer Science, Fudan University, Shanghai, 200433 China; 3 The institute of subtropical Agriculture, China Academy of Sciences, 444 Yuandaer Road, Mapoling, Changsha, 410125 China; 4 The Bioinformatics Lab at Changzhou NO. 7 People’s Hospital, Changzhou, Jiangsu, 213011 China

**Keywords:** PPI network, Protein complex, Gene expression, GO annotation

## Abstract

**Background:**

Effectively predicting protein complexes not only helps to understand the structures and functions of proteins and their complexes, but also is useful for diagnosing disease and developing new drugs. Up to now, many methods have been developed to detect complexes by mining dense subgraphs from static protein-protein interaction (PPI) networks, while ignoring the value of other biological information and the dynamic properties of cellular systems.

**Results:**

In this paper, based on our previous works CPredictor and CPredictor2.0, we present a new method for predicting complexes from PPI networks with both gene expression data and protein functional annotations, which is called CPredictor3.0. This new method follows the viewpoint that proteins in the same complex should roughly have similar functions and are active at the same time and place in cellular systems. We first detect active proteins by using gene express data of different time points and cluster proteins by using gene ontology (GO) functional annotations, respectively. Then, for each time point, we do set intersections with one set corresponding to active proteins generated from expression data and the other set corresponding to a protein cluster generated from functional annotations. Each resulting unique set indicates a cluster of proteins that have similar function(s) and are active at that time point. Following that, we map each cluster of active proteins of similar function onto a static PPI network, and get a series of induced connected subgraphs. We treat these subgraphs as candidate complexes. Finally, by expanding and merging these candidate complexes, the predicted complexes are obtained.

We evaluate CPredictor3.0 and compare it with a number of existing methods on several PPI networks and benchmarking complex datasets. The experimental results show that CPredictor3.0 achieves the highest F1-measure, which indicates that CPredictor3.0 outperforms these existing method in overall.

**Conclusion:**

CPredictor3.0 can serve as a promising tool of protein complex prediction.

## Background

Proteins, as the material basis of life, are the ultimate controller and direct performer of life activities, participate almost all of biological functions. Most proteins do not perform biological functions alone, but form protein complexes with others [1]. So to have a more comprehensive and deep understanding of cell compositions and life processes, the identification of protein complexes is very important.

Although biological techniques such as Tandem Affinity Purification with Mass Spectrometry (TAP-MS) [2] can detect protein complex directly, the accuracy is not high. In addition, biological techniques are usually time-consuming and costly. These make biological techniques cannot meet the requirement of post-genome research for handling big biological data.

With the development of high-throughput experimental technologies, PPI data rapidly increase, which provides chance for using computational methods to detect protein complexes. Moreover, computational methods can overcome drawbacks of experimental technologies. PPI networks can be constructed by using PPI data, where nodes and edges represent proteins and interactions between proteins respectively. Empirical studies on PPI networks indicate that there are modular components in these networks [3]. From the view of network topography, these modules are made up of closely related proteins; From the view of biology, these modules aggregate proteins that perform functions together. Thus, protein complexes can be detected by mining the modular structures (i.e., dense subgraphs or subnetworks) from PPI networks.

So far, there have been many researches that put forward different graph clustering methods to detect local dense subgraphs to detect protein complexes from PPI networks [4–9]. These methods are intuitive and straightforward. To overcome the high false-positive and false-negative problems in PPI networks, many studies have attempted to improve the reliability of PPI data by exploiting gene expression data [10, 11] and protein functional annotations [12, 13] to improve the accuracy of protein complex prediction. In addition to dense subgraph mining based approaches, in the past decade some other method have also developed, including the core-attachment structure based methods [14, 15], methods for non-dense junction complexes and small complexes [16, 17], and methods using dynamic PPI networks [18]. In next section, we will present a relatively comprehensive survey on complex prediction.

In this paper, based on our previous works CPredictor [19] and CPredictor2.0 [16, 17], we propose a new method called CPredictor3.0, which considers both dynamic PPI and functional information. First, we use expression data of different time points to detect active proteins at the same time point, meanwhile we cluster proteins by functional annotations such that each cluster contains proteins of similar function(s). Then, we compute protein clusters of similar function(s) and being active at the same time point by set intersection operation with one set corresponding to an active protein set generated by expression data and the other set corresponding to a protein cluster generated from functional annotations. Following that, we map the resulting clusters onto a static PPI network and obtain a series of induced connected subgraphs, which are treated as candidate complexes. Finally, we identify protein complexes by expanding and merging the candidate complexes. Our experimental results validate the effectiveness of CPredictor3.0, which outperforms the existing methods in overall.

## Related work

So far, a variety of computational methods for complex prediction have been proposed. Here, we present a brief survey on the related works by roughly classifying the existing methods into the following types: methods based on local dense subgraphs, methods based on the Core-Attachment Model, methods based on dynamic PPI networks, methods based on supervised learning. Among them, methods based on local dense subgraphs constitute the most part of existing works. Note that this method hierarchy only reflects our view of existing works. There may be other hierarchies of existing works in the literature. And a brief survey, we cannot cover all existing works, but we try our best to present the major existing works.

### Methods based on local dense subgraphs

As one of earliest computational methods of complex prediction, MCODE [4] first weights each protein based on its core-clustering density in the PPI network, then the protein (say *p*) with the largest weight is selected to be a seed node of a primary complex, which is expanded by including other proteins whose weights exceed a pre-set threshold recursively, till there are no more nodes to be added. If there are unprocessed nodes, new complexes will be generated in the way above. Finally, the neighbors of each complexes generated above are included into the complexes if their weights is higher than a pre-set “fluff” parameter. MCL [5] predicts complexes based on random walk in a PPI network, it is a fast and highly scalable clustering method. To simulate random walk, two operators, expansion and inflation are used to manipulate the adjacency matrix iteratively. The aim of those two operators is to separate dense subgraphs out from the network. Protein complexes predicted by this method are non-overlapped. ClusterONE [6] uses a new measure to compute the cohesiveness of one subgraph, and works by seeding and expanding with neighboring nodes. This method performs better than the other methods when it was developed.

As there exists high false-positives and false-negatives in PPI networks. Some methods weight edges of PPI network using PPI network topology, gene expression data and protein function to improve the reliability of PPI networks. DPClus [20] weights each edge according to the number of shared neighbors of the node pair, then the weight of each node is computed by summing the weights of all its edges. Cao et al. [21] treated complex prediction as an optimization problem and built the objective function by considering a variety of topology characteristics, then the genetic algorithm was employed to detect complexes from PPI networks.

In general, proteins that form functional groups have similar gene expression, so some methods weight edges of PPI networks using expression data. MATISEE [10] measures the intensity of interaction of a pair of proteins using the correlation of expression data. Ou-Yang et al. [11] detected protein complexes from signed PPI networks, the sign of each edge is computed using the pearson correlation coefficient of gene expression of the two proteins.

Except for expression data, protein function provides important clue for protein complex detection. SWEMODE [12] proposed by Lubovac weights each edge based on the semantic similarity of the function(s) of two proteins, the weight of each node is given by the weighted clustering coefficients of the nearest neighbors. Cho et al. [13] weighted each edge according to the functional similarity of two nodes, and the weight of each node is the sum of weights of its edges. The flow simulation algorithm is then used to split the flow from the nodes with larger weights. As each flow goes along edges and its influence decay according to the similarity of each node pair it passes, it stops when its influence is less than a certain threshold. Thus, the PPI network is divided into a plurality of subgraphs consisting of proteins connected by flow from the same source protein.

In our previous works CPredictor [19] and CPredictor2.0 [16, 17], we also used protein functional information. But different from the existing methods, we first used protein functional information to cluster proteins, then mapped the clusters onto PPI networks. The difference between CPredictor and CPredictor2.0 lies in the usage of functional information. In this paper, we follow the same idea of CPredictor and CPredictor2.0, but we also use expression data. That is, we consider the dynamic property of PPI networks.

### Methods based on Core-Attachment model

Gavin et al. [1] studied the structures of yeast protein complexes and found that each protein complex consists of two parts: the core is made of proteins connected tightly, and attachments that have relatively sparse interactions with the core.

Following the core-attachment structure, two methods CORE [14] and COACH [15] were proposed. CORE assesses the probability that two proteins belong to the same core using their common neighbors. Then, larger cores are produced by merging cores of sizes two, three and so on repeatedly. Finally, a protein can be added into one core as attachment if it has interactions with more than half proteins in the core. COACH first identifies small dense subgraphs around proteins of high weight, and then generates cores by merging those dense subgraphs. It uses the same way to add attachments as CORE does. Later, Peng et al. [22] porposed the WPNCA method, which divides a weighted PPI network into multiple closely connected subgraphs by using the PageRank-Nibble algorithm, and then, protein complexes are generated in each subgraph based on the Core-Attachment structure.

### Methods based on dynamic PPI networks

Earlier methods detect complexes from static PPI networks. Actually, the interactions among proteins are dynamic and change over time at different biological stages. In recent year, there are some works on detecting protein complexes from dynamic PPI networks. Tang et al. [18] applied a fixed threshold to cluster proteins using expression data such that each cluster consists of proteins active at the same time point. Since the expression levels of different proteins are quite different, it is unreasonable to use a fixed threshold for all proteins. Later, Wang et al. [23] proposed the three-sigma model to calculate active threshold for proteins, and achieved better performance of complex prediction. Zhang et al. [24] first identified transient and stable protein interactions to construct dynamic PPI networks based on the three-sigma model, then predicted protein complexes from the dynamic PPI networks. Lei et al. [25] constructed dynamic PPI networks using the same method as in [24], and then optimized the parameters of Markov clustering by the firefly algorithm to detect protein complexes.

### Methods based on supervised learning

Some works use supervised learning to detect protein complexes. Qi et al. [26] classified the topological properties of protein complexes into four categories, and used these properties as features to train probability bayesian network, which was used to predict complexes from the subgraphs generated from PPI networks randomly. Yong et al. [27] used true complexes as training data, and a variety of information such as interactions, functions, text and topology as features, to train Bayesian model to predict the probabilities of protein interactions included in small complexes, large complexes and non-complexes, and then small complexes of size 2,3 were extracted.

## Methods

Here, we first give an overview of CPredictor3.0, then describe the major components of CPredictor3.0 in detail.

### Overview

**Fig. 1:**
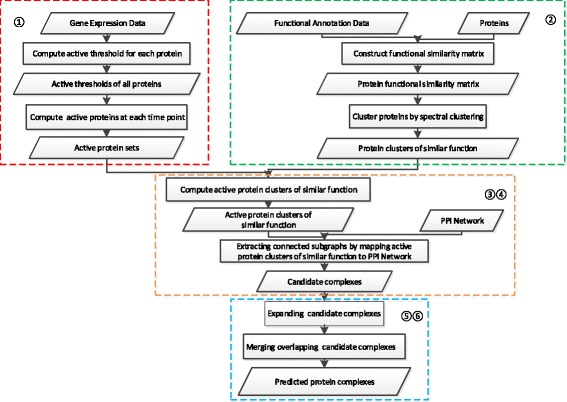
The flowchart of CPredictor3.0. 1) Detecting active proteins; 2) Clustering proteins by function; 3) Computing active proteins of similar function; 4) Extracting candidate complexes from PPI networks; 5) Expanding candidate complexes; 6) Merging candidate complexes

Figure 1 shows the flowchart of CPredictor3.0, which consists of six major steps: 1) Detecting active proteins; 2) Clustering proteins by function; 3) Computing active proteins of similar function; 4) Extracting candidate complexes from PPI networks; 5) Expanding candidate complexes; 6) Merging candidate complexes.

The rationale behind our method is that proteins of a complex performs some function(s) by interacting with each other at the same time and the same place in cellular systems [28]. CPredictor3.0 works like this: First, it detect active proteins from gene expression data for different time points, then it cluster proteins according to functions by using functional annotations. With the results of the above two steps, it computes active protein clusters of similar function. Following that, these clusters are mapped onto a PPI network to extract induced connected subgraphs, which are taken as candidate complexes. Finally, we expand the resulting candidate complexes and merge overlapping ones to get the final predicted complexes. In what follows, we describes these steps in detail.

### Detecting active proteins

Gene expression data reveal the dynamic properties of proteins in their lifetime. As a protein is not always active, its expression level changes with its activity degree. Concretely, higher gene expression level means higher activity. To get the active time points of each protein, Tang et al. [18] set a global fixed threshold for all proteins. There are two drawbacks with a global threshold. On the one hand, there is noise in biological data. On the other hand, the gene expression curve for each protein is different. To solve these problems, Wang et al. [23] proposed the three-sigma model to compute active threshold for each protein. In this paper, we use the three-sigma model to calculate the active threshold for each protein.

Suppose the expression data are measured at *n* time points. For a protein *p*, *V*_*k*_(*p*) represents protein *p*’s expression value at time point *k*, *μ*(*p*) and *σ*(*p*) are the mean and the standard deviation of expression values over the period from 1 to *n*. The active threshold of protein *p* is evaluated as follows:

1 $$ Active(p) = \mu(p) + \beta*\sigma(p)*\left(1-\frac{1}{1+{\sigma(p)}^{2}}\right),  $$

Above, *β* is an adjustable parameter that helps us to get the most optimal threshold. Usually, we set *β*=0, 1, 2, 3.

After obtaining the active thresholds for all proteins, we can collect all active proteins at each time point. That is, for each protein *p* at time point *i* (*i*=1,…,*n*), if its expression value is no less than *Active*(*p*), then it is an active protein at time point *i*. In such a way, we can get the set of active proteins *AP*_*i*_ for each time point *i*. Thus, we have a series of active protein sets { *AP*_*i*_ (*i*=1, …, *n*)}.

### Clustering proteins by function

Here, we cluster proteins by functional annotations. First, we compute the functional similarity of any two proteins using the method proposed by Wang et al. [29], then we employ the spectral clustering algorithm to cluster the proteins with the computed similarity matrix.

The similarity between any two proteins is computed by the GO terms annotated on the two proteins. GO includes a series of biological terms to describe gene and gene products such as protein, and covers three aspects: biological process (BP), cellular component (CC), and molecular function (MF). Here, we use only BP data. GO can be represented as a directed acyclic graph (DAG), in which nodes and edges represent terms and their relationships (e.g. ‘is-a’ and ‘part-of’) between two terms. A GO term *A* can be described as *DAG*_*A*_=(*A,T*_*A*_,*E*_*A*_) where *T*_*A*_ consists of term *A* and all its ancestors in DAG, *E*_*A*_ is composed of all edges (relationships) connecting *A* to all terms in *T*_*A*_. As defined in Wang’s method, the semantic content of a term is the sum of semantic contributions of all its ancestors in *DAG*_*A*_ to *A*. The semantic contribution of term *t* to *A* is as follows:

2 $$ S_{A}(t) = \left\{ \begin{array}{rcl} 1 & &{t=A} \\ max\{w_{e} *S_{A}(t')|t' \in childrenof(t)\} & &{ t \ne A} \end{array} \right.  $$

where function *childrenof*(*t*) returns the children of *t* in *DAG*_*A*_, and *w*_*e*_ as the weight on the edge between *t* and *t* ^′^, which depends on the relationship type between the two terms. For example, the weight is 0.8 for ‘is-a’ and 0.6 for ‘part-of’.

So the semantic value *SV*(*A*) of term *A* is evaluated as follows:

3 $$ SV(A) = \sum_{t \in T_{A}}S_{A}(t).  $$

The semantic similarity *S*_*GO*_(*A,B*) between term *A* and *B* is evaluated as follows:

4 $$ S_{GO}(A,B)= \frac{\sum_{t \in {T_{A} \cap T_{B}}}SV(t)}{SV(A)+SV(B)}.  $$

Generally, one protein may participate one or more biological functions, so one protein may be annotated by multiple terms. For two proteins *P*1 and *P*2, which are annotated by { *go*_11_, *go*_12_, ⋯, *go*_1*m*_} and { *go*_21_, *go*_22_, ⋯, *go*_2*n*_} respectively, their similarity can be evaluated as follows:

5 $$  Sim(P1,P2)= \frac{\sum_{i=1}^{m} Sim\left(go_{1i},P_{2}\right)+\sum_{j=1}^{n} Sim\left(go_{2j},P_{1}\right)}{m+n}  $$

6 $$ Sim(go,P)= max_{1 \leq i \leq k} \left(S_{GO}\left(go, go_{i}\right)\right)  $$

After getting the similarity matrix for all proteins, where each element represents the semantic similarity of two proteins. Then, we apply the spectral clustering algorithm [30] to the matrix to cluster all proteins into *K* disjointed clusters *PC*={ *PC*_1_, *PC*_2_, ⋯, *PC*_*K*_} where *K* is an adjustable parameter to control the number of protein clusters.

### Complex generation

#### Computing active protein clusters of similar function

With the sets of active proteins {*AP*_*i*_|*i*=1,…,*n*} and the set of protein clusters of similar function {*PC*_*j*_|*j*=1,…,*K*}, here we go to compute the active protein clusters of similar function. For time point *i*, the set of active protein clusters of similar function is *APC*_*i*_=*AP*_*i*_∩{*PC*_*j*_|*j*=1,…,*K*}={*AP*_*i*_∩*PC*_*j*_|*j*=1,…,*K*}. Thus, we can get all active protein clusters of similar function as follows:

7 $$ \begin{aligned} APC&=\{APC_{i}|i=1, \cdots, n\}\\ &=\{AP_{i} \cap PC_{j} |i=1, \cdots, n; j=1, \ldots, K\}\\ &=\{APC_{ij}|i=1, \cdots, n; j=1, \ldots, K\}. \end{aligned}  $$

#### Computing candidate complexes

We have already gotten the set of active protein clusters of similar function, considering that complexes consist of interacting proteins, we map all active protein clusters of similar function onto a PPI network *G*=(*V,E*) where *V* and *E* represent proteins and interactions respectively, to get connected subgraphs induced by each cluster on *G*. Concretely, given the active protein cluster of similar function *APC*_*ij*_, we map *APC*_*ij*_ onto *G* and get the induced graph *G*_*ij*_=(*V*_*ij*_,*E*_*ij*_) by *APC*_*ij*_. That is, *V*_*ij*_=*APC*_*ij*_ and *E*_*ij*_ are the set of edges in *G* that connect proteins in *APC*_*ij*_. *G*_*ij*_ may be not a connected graph, i.e., it may consist of several connected subgraphs. We treat each resulting subgraph of size > 1 as a candidate complex. Thus, from *G*_*ij*_ we get a set of candidate complexes *CC*_*ij*_. Similarly, by mapping other active protein clusters of similar function onto *G*, we obtain other candidate complexes. We denote the set of all candidate complexes as *CC* ={*CC*_*ij*_|*i*=1,⋯,*n*;*j*=1,…,*K*}.

#### Candidate complex expanding

Here, we try to expand each candidate complex on *G*. Consider a candidate complex *c*∈*CC*, its corresponding graph is *G*_*c*_=(*V*_*c*_,*E*_*c*_). First, we search the set of neighbor nodes of candidate complex *c* in *G*, which is denoted as *N*_*G*_(*c*). We have

8 $$ N_{G}(c)= \left\{p| E(p,G_{c})\in E \quad and \quad p \in (V-V_{c}) \right\}  $$

where *p* is any protein not in *V*_*c*_ and *E*(*p,G*_*c*_) is the set of interactions between protein *p* and any protein in *G*_*c*_. For any protein *p*∈*N*_*G*_(*c*), if the following condition holds, we add *p* and its interactions with proteins in *c* to *c*:

9 $$ |E(p, G_{c})| \geq \alpha *|V_{c}|.  $$

Above, *α* is a pre-specified threshold. In this paper, it is set to 0.6 by experience. *α* is in the range 0 to 1. When *α* is set to 0, all neighbors of *V*_*c*_ will be added into *V*_*c*_. Otherwise, if *α* is set to 1, no nodes will be added into *V*_*c*_. Usually, if one node interacts with more than half nodes of *V*_*c*_, the node will be added [15, 19]. Our experimental results validate the rationality of the value setting. The expansion process continues till no any more neighbor can be added to *c*. We do expansion to all candidate complexes in *CC*, and denote the set of candidate complexes after expansion as *CC*_*exp*_.

#### Candidate complexes merging

There may be overlapping between candidate complexes in *CC*_*exp*_. For two overlapping candidate complexes, if their overlapping score is larger than a predefined threshold, we merge them to one complex. Concretely, given two candidate complexes *c*_*A*_ and *c*_*B*_, their overlapping score is evaluate as follows:

10 $$ OS(c_{A},c_{B}) = \frac{\left|V_{c_{A}} \cap V_{c_{B}}\right|}{\left|V_{c_{A}} \cup V_{c_{B}}\right|}.  $$

If *OS*(*c*_*A*_,*c*_*B*_)≥*γ*, we merge *c*_*A*_ and *c*_*B*_. Here, *γ* is a pre-specified parameter. By experiments, we set *γ*=0.8. When there are no more candidate complexes that can be merged, the resulting and remaining candidate complexes constitute the final set of predicted complexes.

### The Algorithm

The algorithm of CPredictor3.0 is presented in Algorithm 1. Here, Lines 5-10 are for computing active protein clusters of similar function, Lines 11-24 are for candidate complexes extraction, Lines 25-35 are for candidate complexes expansion, and Lines 36-40 are for candidate complexes merging.



## Results and discussion

### Data sources and Metrics

We downloaded gene expression data GSE3413 [31] from Gene Expression Omnibus (GEO) to compute active proteins. As gene products can cover more than 96% proteins in PPI networks, it is reasonable to detect active proteins from expression data for different time points. GSE3413 is the expression profiling of yeast in the form of matrix, which contains three successive metabolic cycles, and each cycle has 12 time intervals. So each protein has 12 expression values in every cycle. To reduce the impaction of noise, we took the averaged expression values of 12 time points over three cycles, and used these averaged values in our experiments.

In addition to gene expression data, we used three PPI datasets of yeast, which are referred to as Krogan [32], Collins [33] and WI-PHI [34]. The numbers of proteins and interactions in these three datasets are presented in the 2nd and 3rd columns of Table 1. MIPS [35] and CYC2008 [36] were used as reference complex sets, the numbers of complexes and proteins contained in these two sets are presented in the 2nd and 3rd columns of Table 2. In this paper, the GOSemsin package [37] was employed to compute the protein functional similarity matrix.

**Table 1:** The statistics of PPI datasets

PPI network	# proteins	# interactions
Krogan	2674	7075
Collins	1622	9074
WI-PHI	6400	50000

**Table 2:** The statistics of benchmark datasets

benchmark database	# complexes	# proteins
MIPS	313	1237
CYC2008	349	1627

To measure the quality of predicted protein complexes, predicted complexes are checked against with reference complexes. Let *P* =(*V*_*p*_,*E*_*p*_) and *R*= (*V*_*r*_,*E*_*r*_) are a predicted complex and a known complex, respectively. The affinity score (AS) of the two complexes is defined as follows:

11 $$ AS(P,R) = \frac{|V_{p} \cap V_{r}|^{2}}{|V_{p}| *|V_{r}|}.  $$

Usually, *P* and *R* are considered matched when *AS*(*P,R*)≥0.2. This criterion was widely used in the literature [19, 21, 38–43]. However, as stated in PPSampler2 [43], for complexes of size 2, that is, the size of *V*_*p*_ and *V*_*r*_ is 2, then we have $\frac {1}{2 *2} = 0.25 > 0.2$. This means that size-2 candidates can be easily considered as real complexes, which may bring randomness to the final result and affect the correctness of performance evaluation. Actually, most existing methods cannot effectively detect size-2 complexes, because they treat complexes as dense subgraphs while size-2 complexes are just single edges. So a common strategy is simply neglecting the size-2 complexes. In our method, we follow this strategy to discard those predicted complexes with only two proteins.

In our method, *recall*, *precision* and *F1-measure* are used to measure the prediction performance. Let *PS* = { *ps*_1_, ⋯, *ps*_*m*_} and *RS*={ *rs*_1_, ⋯, *rs*_*n*_} are the predicted complex set and the benchmark complex set respectively, the three performance metrics are evaluated as follows:

12 $$\begin{array}{*{20}l} recall &= \frac{N_{r}}{|RS|}, \end{array} $$

13 $$\begin{array}{*{20}l} precision &= \frac{N_{p}}{|PS|}, \end{array} $$

14 $$\begin{array}{*{20}l} F1-measure &= \frac {2*recall *precision}{recall +precision}. \end{array} $$

Above, *N*_*r*_ is the number of reference complexes that match at least one predicted complex, *N*_*p*_ is the number of predicted complexes that match at least one reference complex. |*RS*| and |*PS*| are the size of benchmark complex set and the size predicted complex set respectively.

### Experimental results

We present the experimental results from three aspects. Firstly, we count the size distribution of predicted protein complexes of different algorithms. Secondly, we check the impact of two parameters *K* and *β* on prediction performance of our method. Finally, we compare our method with major existing methods in terms of recall, precision and F1-measure.

#### The size distribution of predicted protein complexes

**Fig. 2:**
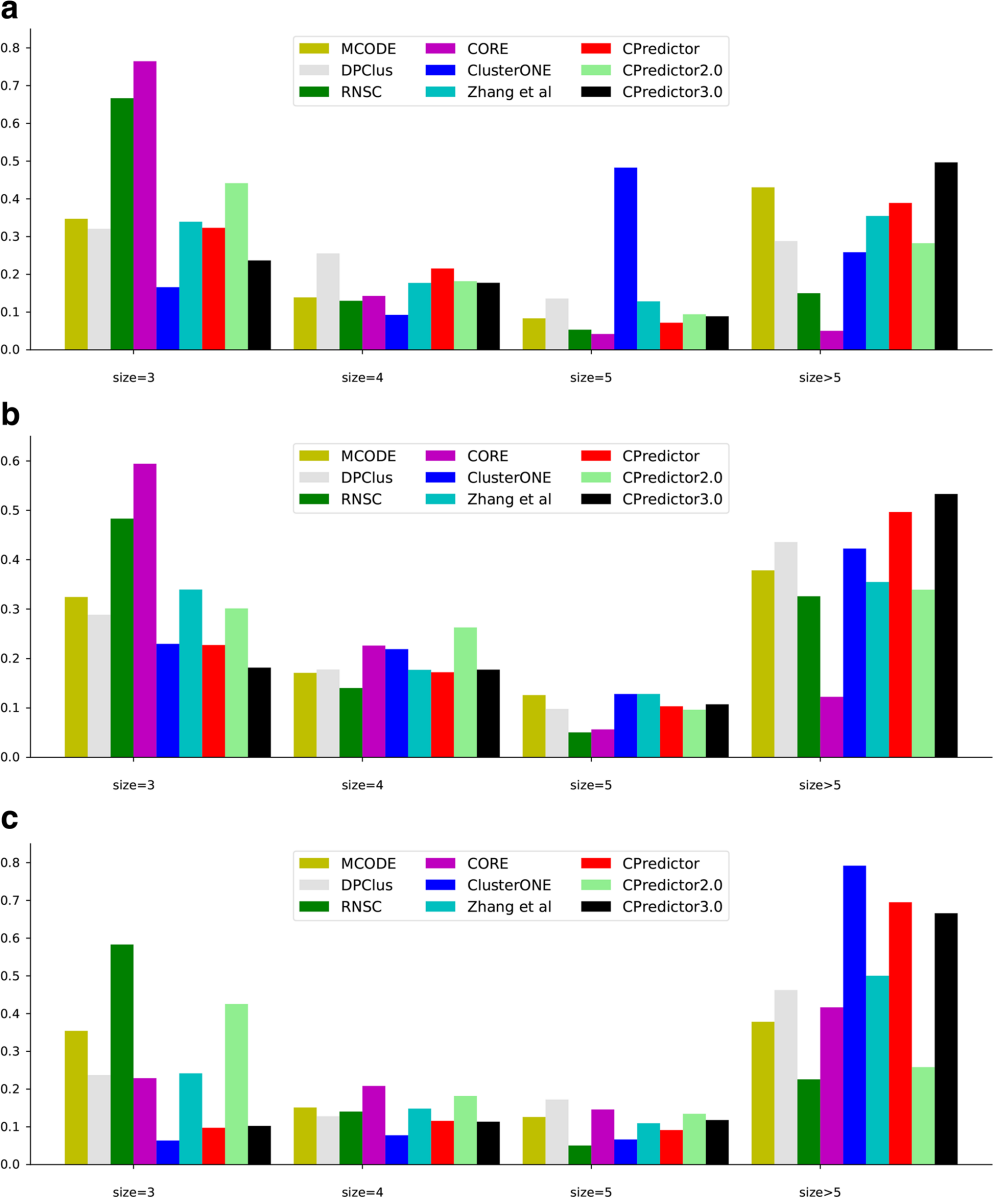
The distribution of protein complex size. **a** Krogan PPI data set. **b** Collins PPI data set. **c** WI-PHI PPI data set

As our method employs function and expression constraints to filter complexes, which may tend to produce small complex candidates. However, our method also use cluster expansion and merging strategies to generate the final predictions. To check the effectiveness of the expansion and merging strategies, here we present the size distribution of predicted protein complexes for different methods on different PPI datasets against different complex benchmark sets in Fig. 2. It is clear that complexes with 5 or more proteins count the largest part of our method’s prediction results. This means that the expansion and merging strategies employed in our method are effective.

#### The effect of parameters on the performance of CPredictor3.0

**Fig. 3:**
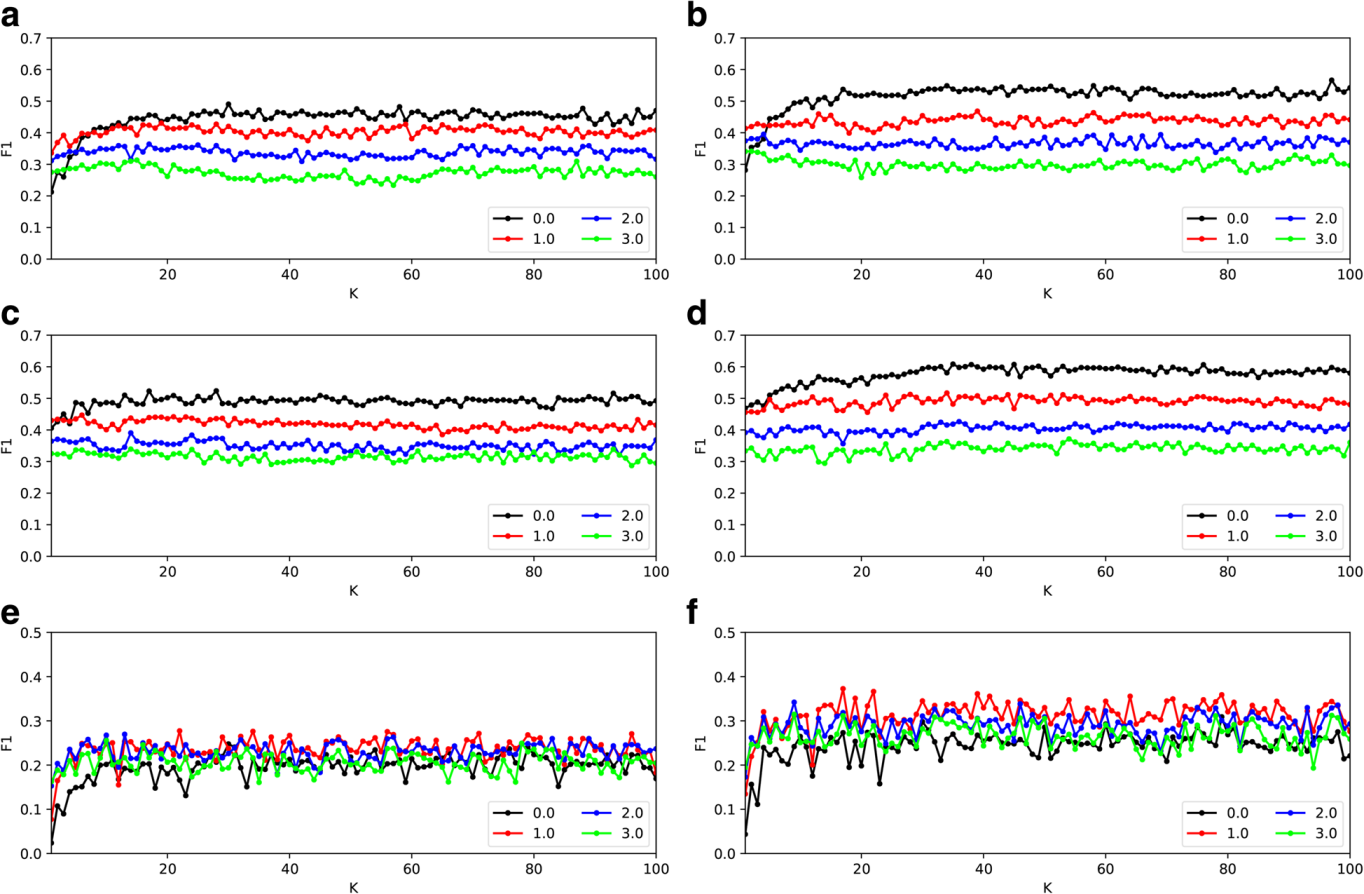
The effect of *K* and *β* on prediction performance. **a** Krogan PPI data and MIPS reference complexes set. **b** Krogan PPI data and CYC2008 reference complexes set. **c** Collins PPI data and MIPS reference complexes set. **d** Collins PPI data and CYC2008 reference complexes set. **e** WI-PHI PPI data and MIPS reference complexes set. **f** WI-PHI PPI data and CYC2008 reference complexes set

In our method, there are two adjustable parameters *K* and *β* which can impact prediction performance. Here, we present the results of how F1-measure changes with the values of the two parameters, which are shown in Fig. 3.

By checking the complexes in the reference sets, we can see that the size of most protein complexes is less than 30. In experiments, we set the value of *K* to from 1 to 100. The parameter *β* is used to set the threshold for filtering active proteins. According to three sigma(SD) model, we set the largest value of *β* to 3, and change it from 0 to 3. As shown in Fig. 3, the performance tends to be stable when *K* is greater than 20. For different *K* values, the best F1-measure is achieved when *β* is set to 0. So in the comparison experiments, we set *K* =30, *β*=0 for Collins PPI data, *K*=35, *β*=0 for Krogan PPI data, and *K*=17, *β*=1 for WI-PHI PPI data.

By checking the predicted complexes further, we can see that there are some large complexes of size > 100 when *K* is set small. This is reasonable. As parameter *K* indicates the number of clusters that the proteins are to be divided. So, small *K* will lead to large clusters, i,e, large complexes, and vice versa.

As for parameter *β*, which is the threshold for filtering active proteins from gene expression data. A larger *β* will results in more proteins being filtered as inactive proteins. In Fig. 3, we can see that when *β* is set to 0, i.e., we set *β* to the mean of expression values over all time points, we get the best performance on Collins and Krogan PPI networks, while the best performance is achieved on WI-PHI network with *β*=1.0.

#### Comparison with major existing methods

Here, we compare our method CPreditor3.0 with eight existing protein complex prediction methods, including MCODE [4], DPClus [20], RNSC [44], CORE [14], ClusterONE [6], Zhang et al. [24], CPredictor [19], and CPredictor2.0 [16, 17]. Some of them are the state of the art techniques, such as ClusterONE [6] and CPredictor2.0 [16, 17]. All parameters in these compared methods were set as suggested by their authors.

**Fig. 4:**
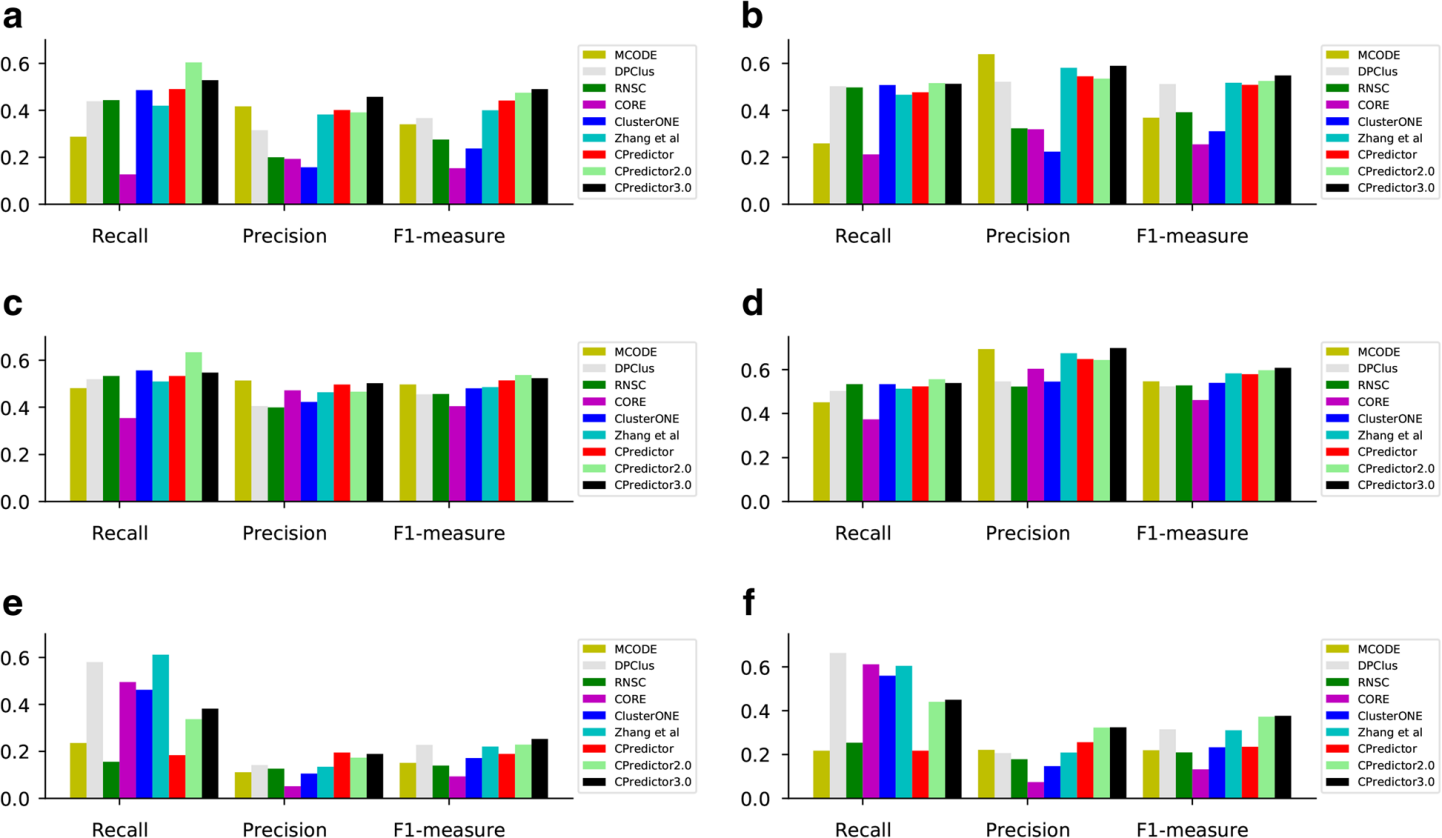
Performance comparison with eight existing protein complex prediction algorithms in terms of recall, precision, and F1-measure. Our method CPreditor3.0 achieves the highest F1-measure in five of the six experimental settings. (**a**) Results with Krogan as PPI dataset and MIPS as complex reference set, (**b**) Results with Krogan as PPI dataset and CYC2008 as complex reference set, (**c**) Results with Collins as PPI dataset and MIPS as complex reference set, (**d**) Results with Collins as PPI dataset and CYC2008 as complex reference set, (**e**) Results with WI-PHI as PPI dataset and MIPS as complex reference set, (**f**) Results with WI-PHI as PPI dataset and CYC2008 as complex reference set

The experimental results are shown in Fig. 4. We can see in five of the six experimental settings, CPredictor3.0 achieves the highest F1-measure. And in the remaining setting, CPredictor3.0 still has comparable F1-measure to the best one. In three of the six settings, CPredictor3.0 has the highest precision, and has the 2nd highest precision in the other three settings. As for recall, CPredictor3.0 stays at the second or third position in five settings and at the fifth position in one setting. Thus, in overall our method performs best among the nine methods.

From Fig. 4, we can see that all methods have different performance on different PPI datasets and complexes reference sets. To give a detailed picture, we compute the average F1 values of all compared methods in the six settings. The results are presented in Table 3. Checking these results, we can see that: on the one hand, giving the PPI dataset (Krogan, Collins or WI-PHI), the performance with CYC2008 as reference set is better than that with MIPS as reference set. On the other hand, giving the complex reference set (MIPS or CYC2008), using the Collins PPI dataset gets the best performance and using the WI-PHI PPI dataset has the worst performance. This observation can be explained by the number of overlapping proteins between the PPI dataset and the reference set used in the prediction. Comparing with Krogan and Collins, WI-PHI has the largest number of proteins. Most predicted complexes from WI-PHI cannot find matching complexes in the two reference sets, which results in low performance.

**Table 3:** The average F1-measure values of the nine algorithms on various PPI datasets and complexes reference sets

	Collins		Krogan		WI-PHI	
	CYC2008	MIPS	CYC2008	MIPS	CYC2008	MIPS
F1	0.5518	0.4837	0.4376	0.3534	0.2672	0.1861

To give a detailed explanation, we compute the ratio of the number of overlapping proteins between each PPI dataset and each complexs reference set over the number of proteins contained in the PPI dataset. We call it “overlapping ratio” in short. The results are presented in Table 4. From this table, we can see that 30.6% and 49.2% proteins in the Krogan PPI dataset and the Collins PPI dataset are overlapping with that of the MIPS complex set, while there are only 19.1% proteins in the WI-PHI PPI dataset are overlapping with that of the MIPS complex set. The overlapping ratio of Krogan, Collins and WI-PHI with CYC2008 are 43.1, 68.8 and 25.3% respectively. In summary, for any PPI dataset, the overlapping ratio with CYC2008 is higher than that with MIPS; For any reference set, the highest overlapping ratio is with Collins, then with Krogan, and the lowest overlapping ratio is with WI-PHI. This trend is completely consistent with the results in Table 3. This explains the performance difference of the six settings.

**Table 4:** Overlapping protein ratios of between PPI datasets and complexes reference sets

Benchmark database	Krogan	Collins	WI-PHI
MIPS	30.6%	49.2%	19.1%
CYC2008	43.1%	68.8%	25.3%

## Conclusions

This paper introduced a new method CPredictor3.0 to boost complex prediction performance from PPI networks by using both expression data and functional annotations. Experiments on three commonly used PPI datasets and two benchmark complexes sets show that CPreditor3.0 performs best in overall. It is well recognized that complexes consist of proteins that have similar function and are active at the same time and place in cellular systems. Our method considers all these aspects, including function and dynamic interaction by using PPI data, functional annotations and expression data. This may explain the best performance of our method.

As for future work, on the one hand, we are considering more advanced models to extract complexes from PPI networks, such as graph sparsity models [45] and temporal graph mining models [46]. On the other hand, small complex detection is a more challenging task [17], which is another focus of our future study. Thirdly, for better complex prediction performance, we will also consider building reliable and robust PPI networks by fusing multiple networks [47].

## Abbreviations

GO: Gene ontology
DAG: Directed acyclic graph
PPI: Protein-protein interaction

## References

[1] Gavin AC, Aloy P, Grandi P, Krause R, Boesche M, Marzioch M, Rau C, Jensen LJ, Bastuck S, Dumpelfeld B, Edelmann A, Heurtier MA, Hoffman V, Hoefert C, Klein K, Hudak M, Michon AM, Schelder M, Schirle M, Remor M, Rudi T, Hooper S, Bauer A, Bouwmeester T, Casari G, Drewes G, Neubauer G, Rick JM, Kuster B, Bork P, Russell RB, Superti-Furga G (2006). Proteome survey reveals modularity of the yeast cell machinery. Nature.

[2] Rigaut G, Shevchenko A, Rutz B, Wilm M, Mann M, Seraphin B (1999). A generic protein purification method for protein complex characterization and proteome exploration. Nat Biotechnol.

[3] Barabasi AL, Oltvai ZN (2004). Network biology: Understanding the cell’s functional organization. Nat Rev Genet.

[4] Bader GD, Hogue CW (2003). An automated method for finding molecular complexes in large protein interaction networks. BMC Bioinformatics.

[5] Pereira-Leal JB, Enright AJ, Ouzounis CA (2004). Detection of functional modules from protein interaction networks. Proteins Struct Funct Bioinforma.

[6] Nepusz T, Yu H, Paccanaro A (2012). Detecting overlapping protein complexes in protein-protein interaction networks. Nat Methods.

[7] Spirin V, Mirny LA (2003). Protein complexes and functional modules in molecular networks. Proc Natl Acad Sci.

[8] Adamcsek B, Palla G, Farkas IJ, Derenyi I, Vicsek T (2006). Cfinder: locating cliques and overlapping modules in biological networks. Bioinformatics.

[9] Zhang W, Zou X (2015). A new method for detecting protein complexes based on the three node cliques. IEEE/ACM Trans Comput Biol Bioinforma.

[10] Ulitsky I, Shamir R (2007). Identification of functional modules using network topology and high-throughput data. BMC Syst Biol.

[11] Ou-Yang L, Dai DQ, Zhang XF (2015). Detecting protein complexes from signed protein-protein interaction networks. IEEE/ACM Trans Comput Biol Bioinforma.

[12] Lubovac Z, Gamalielsson J, Olsson B (2006). Combining functional and topological properties to identify core modules in protein interaction networks. Proteins Struct Funct Bioinforma.

[13] Cho YR, Hwang W, Ramanathan M, Zhang A (2007). Semantic integration to identify overlapping functional modules in protein interaction networks. BMC Bioinformatics.

[14] Leung HCM, Xiang Q, Yiu SM, Chin FYL (2009). Predicting protein complexes from ppi data: A core-attachment approach. J Comput Biol.

[15] Wu M, Li X, Kwoh CK, Ng SK (2009). A core-attachment based method to detect protein complexes in ppi networks. BMC Bioinformatics.

[16] Xu B, Guan J, Wang Y, Zhou S, Bourgeois A, Skums P, Wan X, Zelikovsky A (2016). Cpredictor2.0: Effectively detecting both small and large complexes from protein-protein interaction networks. Lecture Notes in Bioinformatics. vol. 9683.

[17] Xu B, Wang Y, Wang Z, Zhou J, Zhou S, Guan J (2017). An effective approach to detecting both small and large complexes from protein-protein interaction networks. BMC Bioinformatics.

[18] Tang X, Wang J, Liu B, Li M, Chen G (2011). A comparison of the functional modules identified from time course and static ppi network data. BMC Bioinformatics.

[19] Xu B, Guan J (2014). From function to interaction: A new paradigm for accurately predicting protein complexes based on protein-to-protein interaction networks. IEEE/ACM Trans Comput Biol Bioinforma.

[20] Altaf-Ul-Amin M, Shinbo Y, Mihara K, Kurokawa K, Kanaya S (2006). Development and implementation of an algorithm for detection of protein complexes in large interaction networks. BMC Bioinformatics.

[21] Cao B, Luo J, Liang C, Wang S, Song D (2015). Moepga: A novel method to detect protein complexes in yeast protein–protein interaction networks based on multiobjective evolutionary programming genetic algorithm. Comput Biol Chem.

[22] Peng W, Wang J, Zhao B, Wang L (2015). Identification of protein complexes using weighted pagerank-nibble algorithm and core-attachment structure. IEEE/ACM Trans Comput Biol Bioinforma (TCBB).

[23] Wang J, Peng X, Li M, Pan Y (2013). Construction and application of dynamic protein interaction network based on time course gene expression data. Proteomics.

[24] Zhang Y, Lin H, Yang Z, Wang J, Liu Y, Sang S (2016). A method for predicting protein complex in dynamic ppi networks. BMC Bioinformatics.

[25] Lei X, Wang F, Wu FX, Zhang A, Pedrycz W (2016). Protein complex identification through markov clustering with firefly algorithm on dynamic protein–protein interaction networks. Inf Sci.

[26] Qi Y, Balem F, Faloutsos C, Klein-Seetharaman J, Bar-Joseph Z (2008). Protein complex identification by supervised graph local clustering. Bioinformatics.

[27] Yong CH, Maruyama O, Wong L (2014). Discovery of small protein complexes from ppi networks with size-specific supervised weighting. BMC Syst Biol.

[28] Ruepp A, Brauner B, Dunger-Kaltenbach I, Frishman G, Montrone C, Stransky M, Waegele B, Schmidt T, Doudieu ON, Stumpflen V, Mewes HW (2007). Corum: the comprehensive resource of mammalian protein complexes. Nucleic Acids Res.

[29] Wang JZ, Du Z, Payattakool R, Yu PS, Chen CF (2007). A new method to measure the semantic similarity of go terms. Bioinformatics.

[30] von Luxburg U (2007). A tutorial on spectral clustering. Stat Comput.

[31] Tu BP, Kudlicki A, Rowicka M, McKnight SL (2005). Logic of the yeast metabolic cycle: Temporal compartmentalization of cellular processes. Science.

[32] Krogan NJ, Cagney G, Yu HY, Zhong GQ, Guo XH, Ignatchenko A, Li J, Pu SY, Datta N, Tikuisis AP, Punna T, Peregrin-Alvarez JM, Shales M, Zhang X, Davey M, Robinson MD, Paccanaro A, Bray JE, Sheung A, Beattie B, Richards DP, Canadien V, Lalev A, Mena F, Wong P, Starostine A, Canete MM, Vlasblom J, Wu S, Orsi C, Collins SR, Chandran S, Haw R, Rilstone JJ, Gandi K, Thompson NJ, Musso G, St Onge P, Ghanny S, Lam M, Butland G, Altaf-Ui AM, Kanaya S, Shilatifard A, O’Shea E, Weissman JS, Ingles CJ, Hughes TR, Parkinson J, Gerstein M, Wodak SJ, Emili A, Greenblatt JF (2006). Global landscape of protein complexes in the yeast saccharomyces cerevisiae. Nature.

[33] Collins SR, Kemmeren P, Zhao XC, Greenblatt JF, Spencer F, Holstege FCP, Weissman JS, Krogan NJ (2007). Toward a comprehensive atlas of the physical interactome of saccharomyces cerevisiae. Mol Cell Proteomics.

[34] Kiemer L, Costa S, Ueffing M, Cesareni G (2007). Wi-phi: a weighted yeast interactome enriched for direct physical interactions. Proteomics.

[35] Mewes HW, Amid C, Arnold R, Frishman D, Guldener U, Mannhaupt G, Munsterkotter M, Pagel P, Strack N, Stumpflen V, Warfsmann J, Ruepp A (2004). Mips: analysis and annotation of proteins from whole genomes. Nucleic Acids Res.

[36] Pu S, Wong J, Turner B, Cho E, Wodak SJ (2009). Up-to-date catalogues of yeast protein complexes. Nucleic Acids Res.

[37] Yu G, Li F, Qin Y, Bo X, Wu Y, Wang S (2010). Gosemsim: an r package for measuring semantic similarity among go terms and gene products. Bioinformatics.

[38] Pellegrini M, Baglioni M, Geraci F (2016). Protein complex prediction for large protein protein interaction networks with the core&peel method. BMC Bioinformatics.

[39] Li X, Wu M, Kwoh CK, Ng SK (2010). Computational approaches for detecting protein complexes from protein interaction networks: a survey. BMC Genomics.

[40] Luo J, Lin D (2015). A cell-core-attachment approach for identifying protein complexes in ppi network. Natural Computation (ICNC), 2015 11th International Conference On.

[41] Hu AL, Chan KC (2013). Utilizing both topological and attribute information for protein complex identification in ppi networks. IEEE/ACM Trans Comput Biol Bioinforma.

[42] Li XL, Foo CS, Tan SH, Ng SK (2005). Interaction graph mining for protein complexes using local clique merging. Genome Inform.

[43] Widita CK, Maruyama O (2013). Ppsampler2: Predicting protein complexes more accurately and efficiently by sampling. BMC Syst Biol.

[44] King AD, Przulj N, Jurisica I (2004). Protein complex prediction via cost-based clustering. Bioinformatics.

[45] Gao L, Zhou S, Schuurmans D, Wellman MP (2016). Group and graph joint sparsity for linked data classification. Proceedings of AAAI.

[46] Yang Y, Yan D, Wu H, Cheng J, Zhou S, Lui JCS, Krishnapuram B, Shah M, Smola AJ, Aggarwal CC, Shen D, Rastogi R (2016). Diversified temporal subgraph pattern mining. Proceedings of KDD.

[47] Zheng X, Wang Y, Tian K, Zhou J, Guan J, Luo L, Zhou S (2017). Fusing multiple protein-protein similarity networks to effectively predict lncrna-protein interactions. BMC Bioinformatics.

